# Supplementary material for: Operando and three-dimensional visualization of anion depletion and lithium growth by stimulated Raman scattering microscopy
Source: Nat Commun. 2018 Jul 30;9:2942. doi: 10.1038/s41467-018-05289-z (PMC6065384; doi:10.1038/s41467-018-05289-z)
Supplement: Supplementary file 1 — Supplementary Information [file 41467_2018_5289_MOESM1_ESM.pdf]

Supplementary information

Operando and Three-Dimensional Visualization of Anion Depletion and Lithium Growth by Stimulated Raman Scattering Microscopy

Cheng et al.

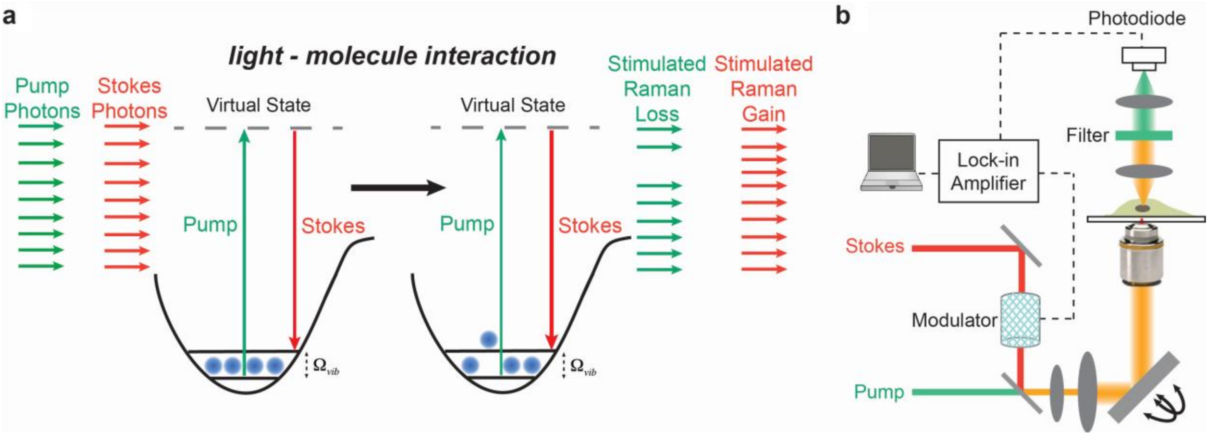

Supplementary Figure 1. Principle and experimental setup for stimulated Raman scattering (SRS) microscopy. **a.** Principle: when the energy difference between the Pump photons and the Stokes photons matches with the vibrational frequency (Ω_{vib}) of the chemical bond of interest, this bond is efficiently driven from vibrational ground state to its vibrational excited state, mediated by a virtual state. Such vibrational excitation results in a photon in Pump beam being annihilated (stimulated Raman loss) and a photon in the Stokes beam being created (stimulated Raman gain). Either the stimulated Raman loss or gain could serve as the contrast for SRS microscopy. **b.** Experimental setup: A pump beam (pulsed, pico-second) and an intensity-modulated Stokes beam (pulsed, pico-second) are both temporally and spatially synchronized before being focused on sampes. The energy difference between the pump and the Stokes photons are tuned into the vibrational frequency for the chemical bond of interest. Selective detection of pump-laser intensity change (i.e., stimulated Raman loss) through a lock-in amplifier targeted at the same frequency as the modulation of the Stokes beam serve as the SRS contrast. By scanning the laser beams across the sample, a chemical image is formed.

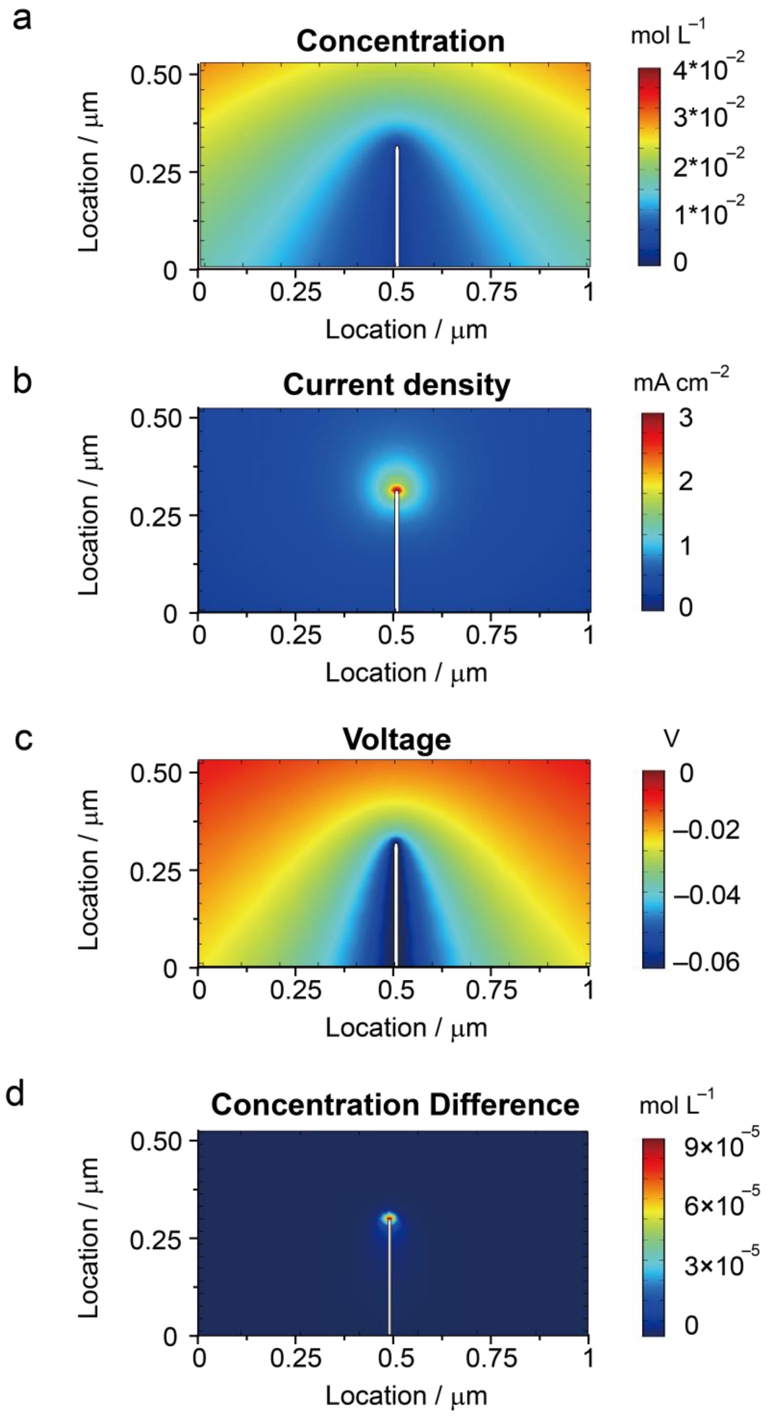

Supplementary Figure 2. COMSOL finite element simulation validating that the electro-neutrality requires the local concentration of cation and anion to be same. Only the tip area was shown here for more clear observation. **a**. The concentration map of Li^+ at depletion. **b**. Current density distribution. **c**. Voltage distribution in the electrolytic cell. **d**. The concentration difference between cation and anion around the electrode tip.

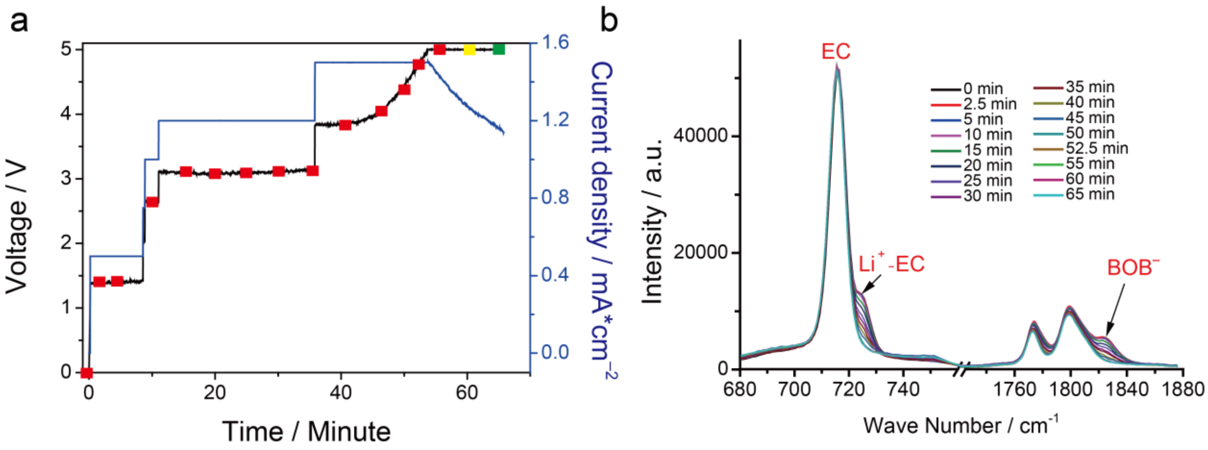

Supplementary Figure 3. Experimental Validation of charge neutrality. **a**. The voltage and current curve versus time plot in the lithium- lithium electrolytic cell used in Fig. 2. The colored square shows the time when Raman spectra were taken, and their corresponding positions can be found in the optical images in Fig. 2c. **b**. Raman Spectra taken from the start of deposition (0 min) to the end of the deposition (65 min). The intensity of both peaks decreases over time. The corresponding concentration changes of Li^{+} and BOB^{-} versus time were shown in Fig. 2d.

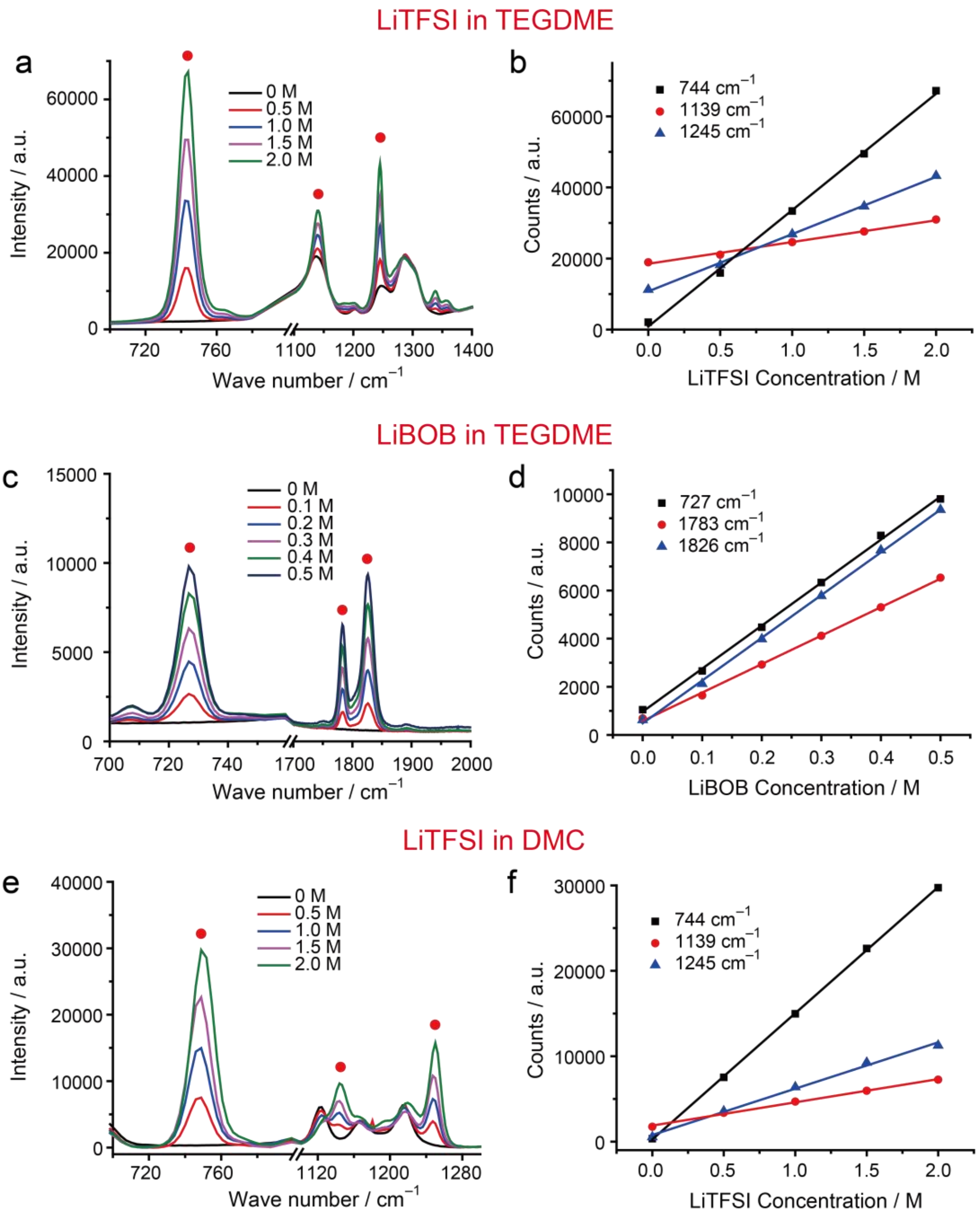

Supplementary Figure 4. Linearity examination. **a**. Spontaneous Raman of LiTFSI in TEGDME at different concentrations. **b**. The linear relationship between Raman intensity and LiTFSI concentration at three wavenumbers. **c**. Spontaneous Raman of LiBOB in TEGDME at different concentrations. **d**. The linear relationship between Raman intensity and LiBOB concentrations at three wavenumbers. **e**. Spontaneous Raman of LiTFSI in DMC at different concentrations. **f**. The linear relationship between Raman intensity and LiTFSI concentration at three wavenumbers. All peaks correspond to anions, and the Raman intensity of each peak in **b**, **d**, and **f** is for a specific wavenumber, but not integral of the peak, to mimic the intensity of single wavenumber detected in our stimulated Raman microscopy.

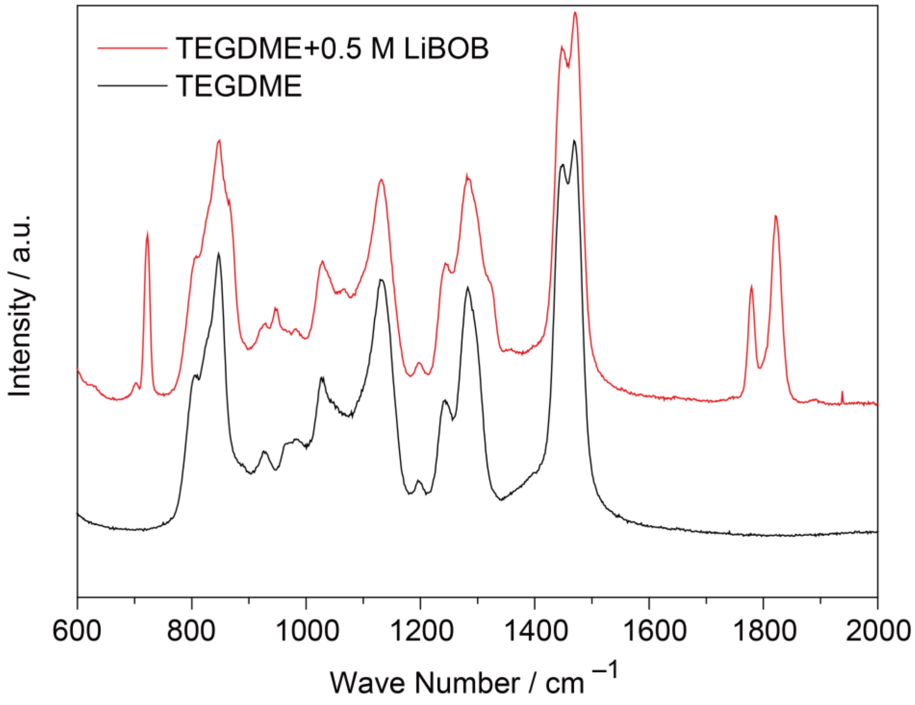

Supplementary Figure 5. Spontaneous Raman of TEGDME and 0.5 M LiBOB/TEGDME. The peak at 1830 cm^{-1} corresponds to C=O bonds in BOB^{-}.

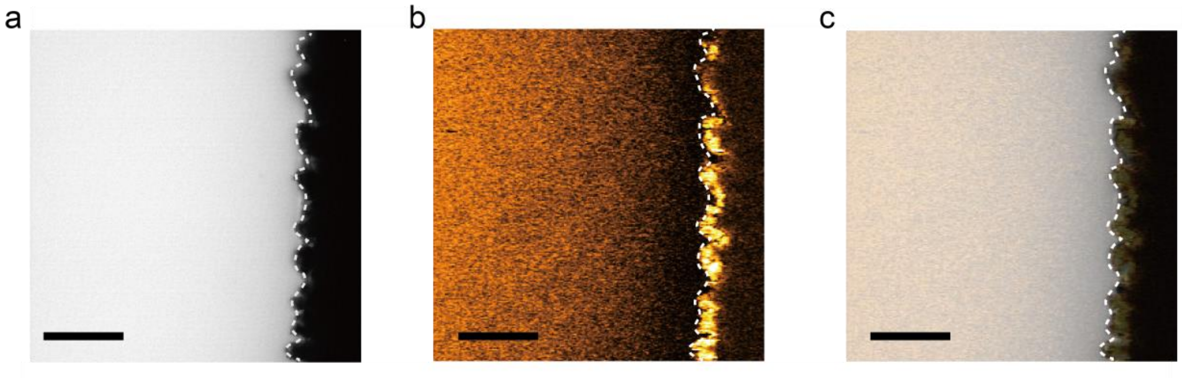

Supplementary Figure 6. The comparison of bright field image and SRS image. Lithium electrode boundaries in **a**. Bright field, **b**. SRS image, and **c**. Overlapped bright field and SRS image are shown. The white contour is the edge of the lithium electrode, showing perfect matching between the SRS image and the bright field image. Scale bars in all images are 100 μm.

a

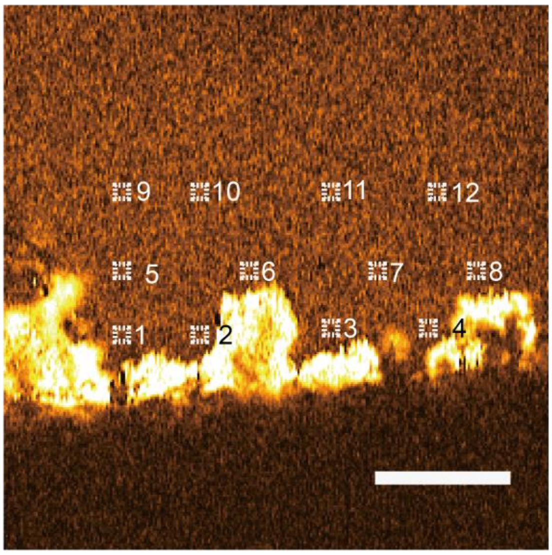

b

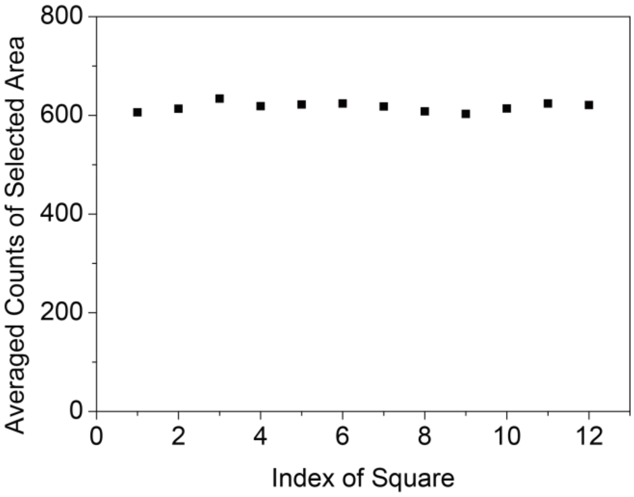

Supplementary Figure 7. An example showing that Raman signal will not be affected by dendrites. **a**. An SRS 2D image exhibiting the Li^{+} concentration distribution after the dendrite growth. The electrode has been rested for 30 minutes so that Li^{+} concentration should be uniform everywhere at 0.33 M. **b**. The average Raman signal of selected area 1–9. Each square has a lateral size of 10 μm, showing the relative position to dendrite has little impact on the Raman signal of the surrounding environment. The scale bar is 100 μm. The average counts and the standard deviation are 617 and 8.8, respectively. The relative standard deviation is 1.42%, corresponding to 4.7 mM.

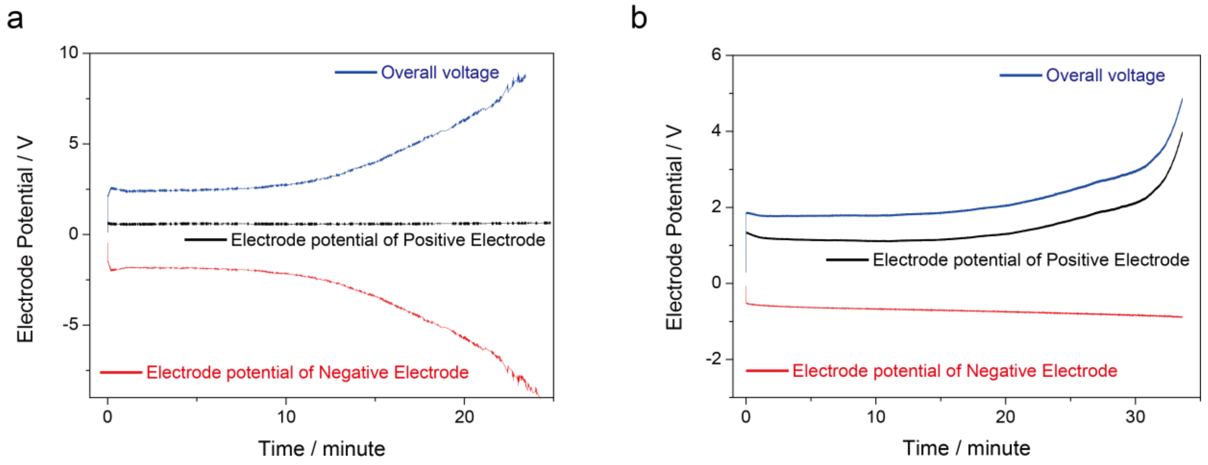

Supplementary Figure 8. Voltage profile of three-electrode cells with different initial concentrations. The concentrations are: **a**. 0.33 M and **b**. 0.5 M LiBOB in TEGDME gel electrolyte. The applied currents for both cells are 1.5 mA cm^{-2}. When 0.33 M LiBOB electrolyte is used, the voltage increase is caused by the depletion of Li ions in the vicinity of the negative electrode (red line in **a**). When 0.5 M LiBOB electrolyte is used, the voltage increase is caused by the accumulation and precipitation of LiBOB in the vicinity of the positive electrode (black line in **b**) instead of ion depletion at the anode.

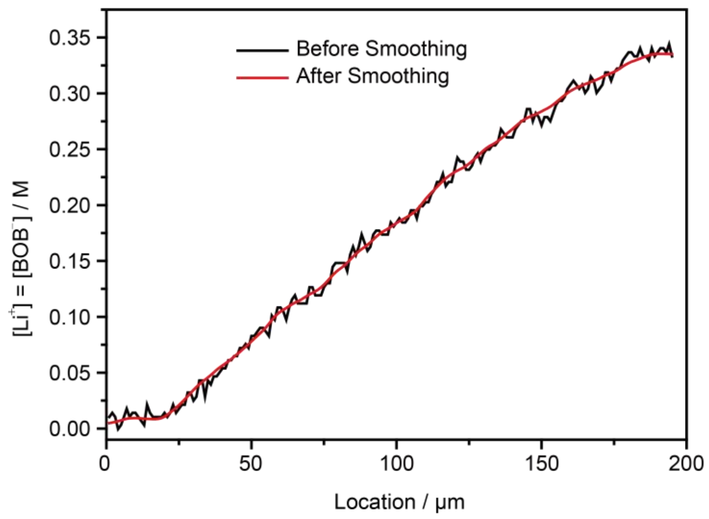

Supplementary Figure 9. An example of kernel smoothing. Details can be found in methods section. The black line is the experimental result before kernel smoothing, and the red line is the result of Kernel fitting showing the reduction of noise.

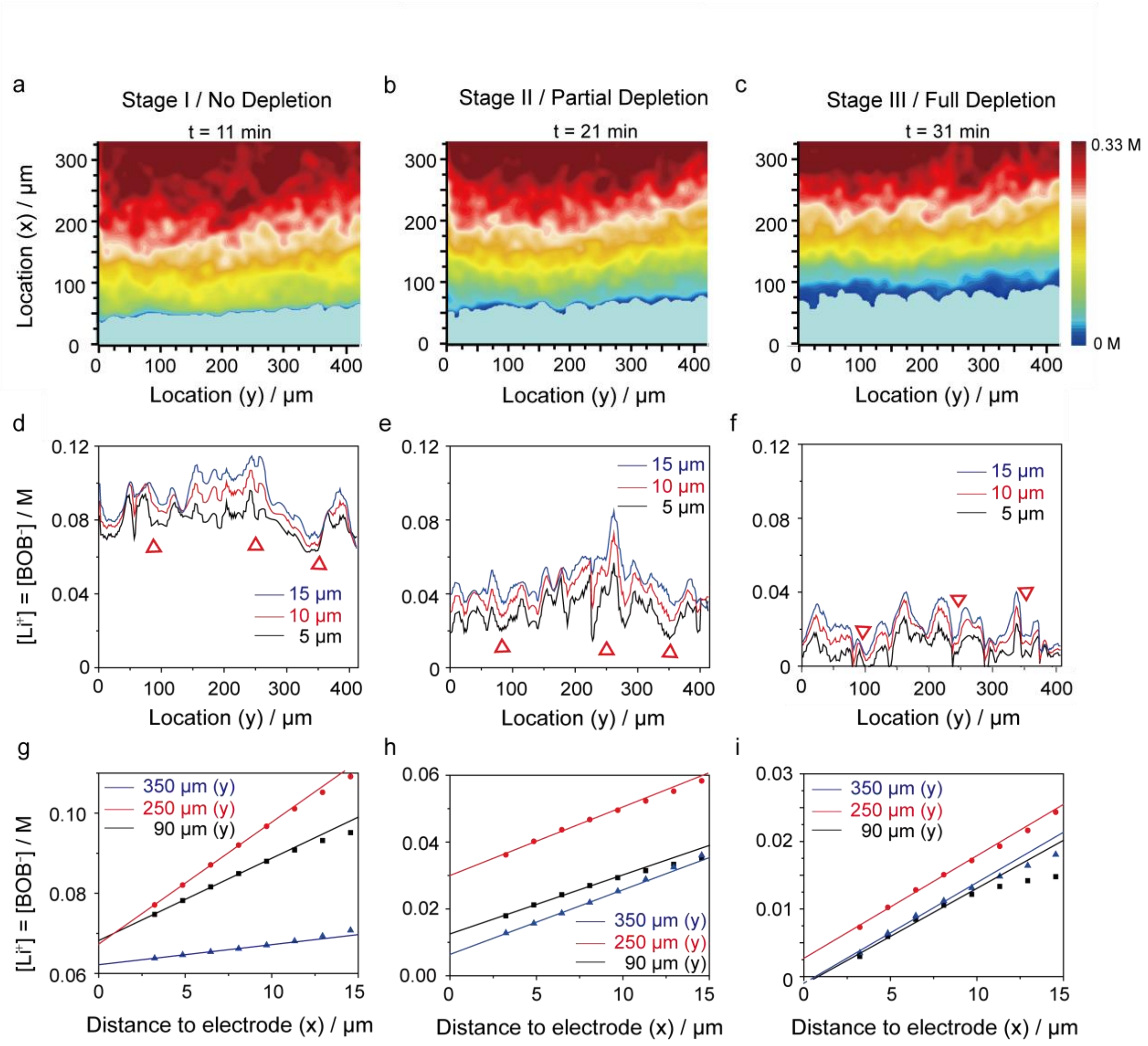

Supplementary Figure 10. Kernel smoothing of 2D SRS images and surface Li^+ concentration ($[Li^+]_{0\mu m}$) deduction. **a**. No depletion at 11 minutes; **b**. Partial depletion at 21 minutes; **c**. Full depletion at 31 minutes. The concentration difference is visualized by a jet colormap, and the color bar is at right. **d**,**e**,**f** show local concentration of Li^+ 5 μm, 10 μm and 15 μm away from surface ($[Li^+]_{5\mu m}$, $[Li^+]_{10\mu m}$ and $[Li^+]_{15\mu m}$). **g**, **h**, **i** show the concentration gradient along the x-direction of three different y locations. The surface concentration ($[Li^+]_{0\mu m}$, Fig. 3h) is deducted from linear extrapolation of data points between 3 and 10 μm by assuming a linear relationship. The detailed procedure of smoothing can be found in the methods section. A representative example is shown in Supplementary Fig. 9.

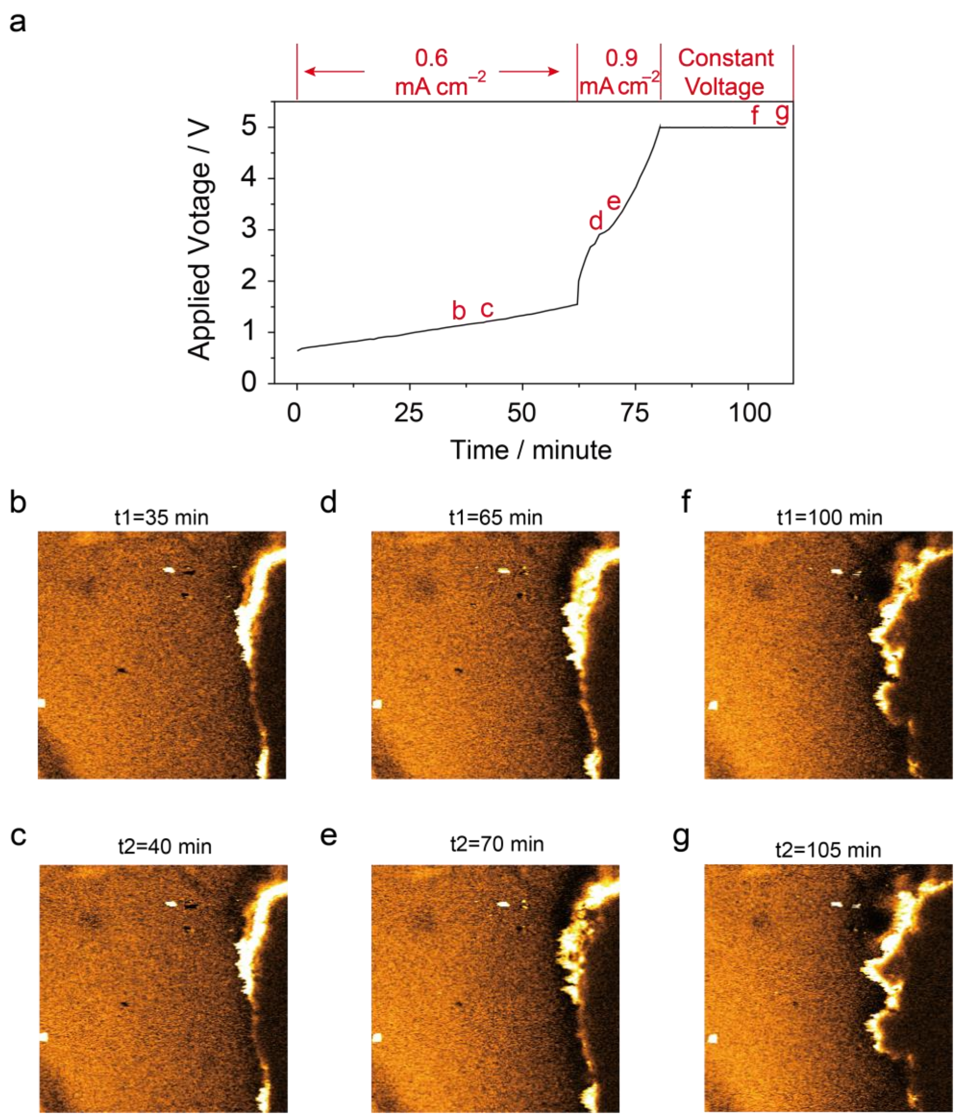

Supplementary Figure 11. Original data for Fig. 4 and Supplementary note 8. **a**. Voltage profile and SRS 2D images of three different cases. **b–g** are the SRS 2D images. **b/c**. No depletion at 35/40 minutes. **d/e**. Partial depletion at 65/70 minutes. **f/g**. Full depletion at 100/105 minutes.

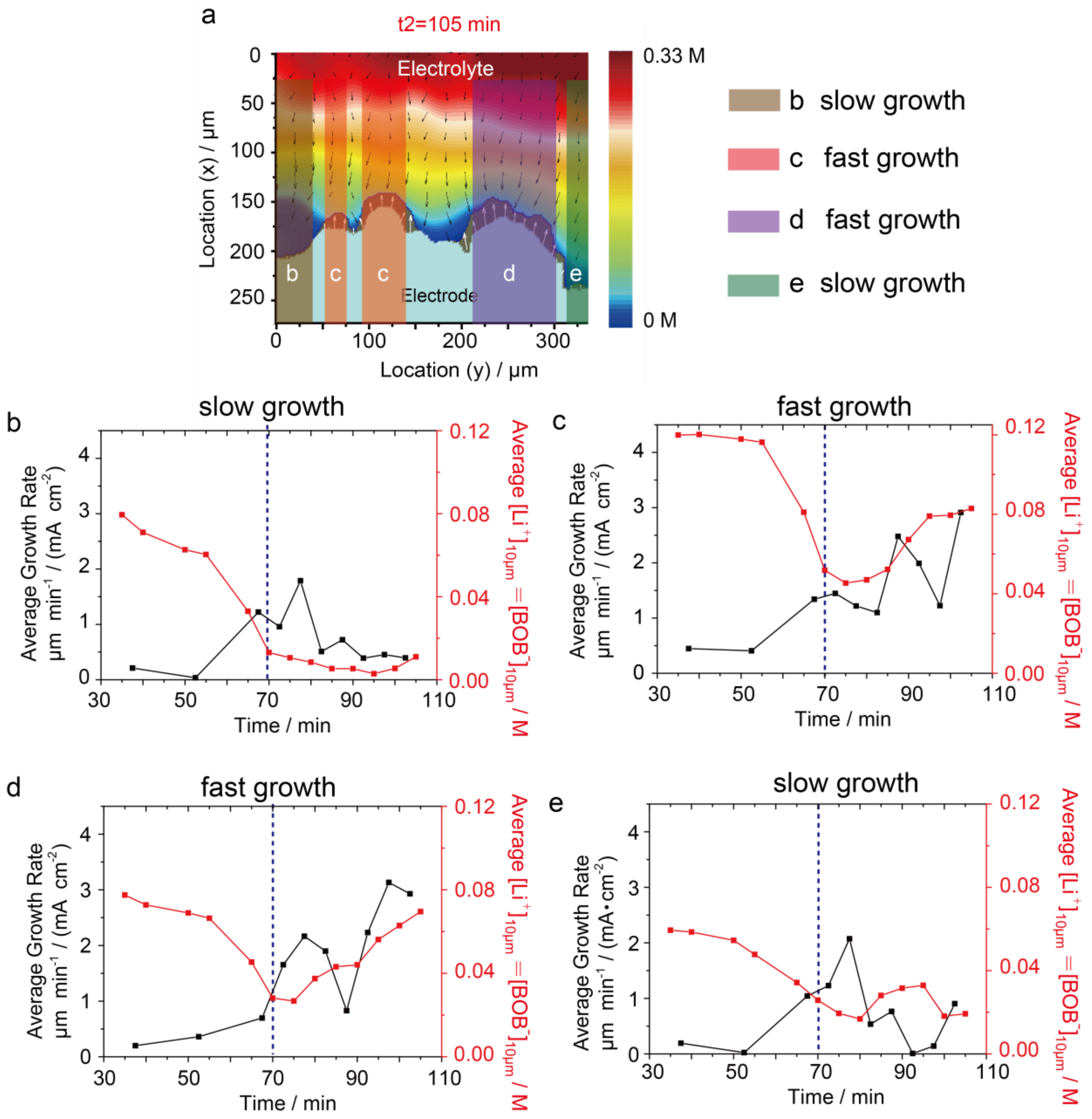

Supplementary Figure 12. Temporal evolution of the Li growth rate and $[Li^+]_{10\mu m}$ at different regions. a. Li electrode and $[Li^+]$ in the electrolyte at T=105 min. This figure is the same as Fig. 4h. Regions of interests are marked as follows. Fast growth: **c** (54 μm–72 μm, 96 μm–142 μm) and **d** (210 μm–300 μm). After depletion starts (blue vertical line), a cooperative increase in growth rate and $[Li^+]_{10\mu m}$ is observed. Slow growth: **b** (0 μm–40 μm) and **e** (312 μm–336 μm). Both growth rate and $[Li^+]_{10\mu m}$ remain at a low level. The blue dashed lines indicate the time when depletion starts.

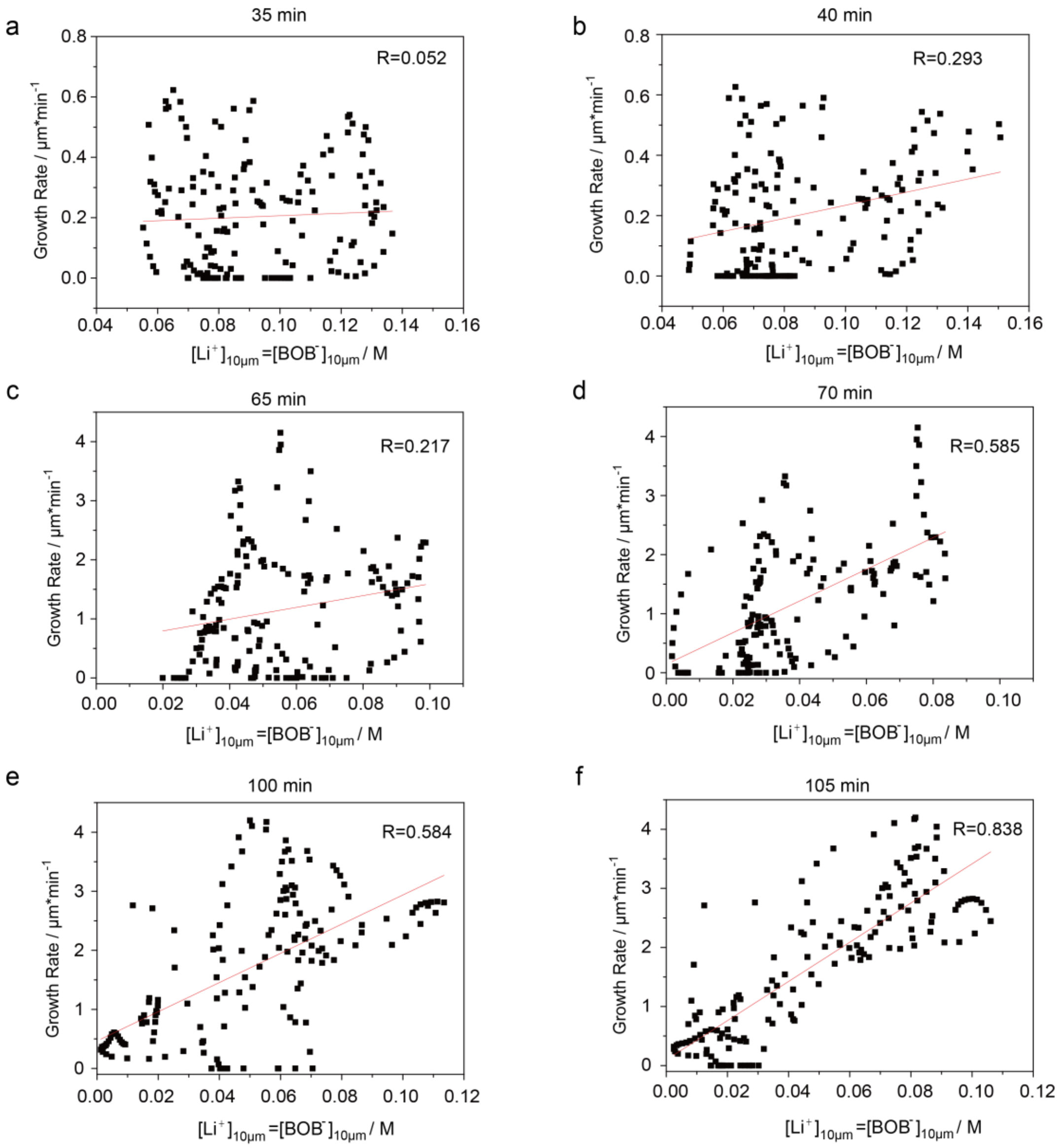

Supplementary Figure 13. The correlation between the local lithium growth rate and $[Li^+]_{10\mu m}$ at t1 / t2. The data are derived from Figure 4. The correlation coefficient is labeled in each plot. **a** and **b**, the correlation between local lithium growth rate and $[Li^+]_{10\mu m}$ at 35 min (**a**) and 40 min (**b**) for stage I; **c** and **d**, the correlation between local lithium growth rate and $[Li^+]_{10\mu m}$ at 65 min (**c**) and 70 min (**d**) at stage II; **e** and **f**, the correlation between local lithium growth rate and $[Li^+]_{10\mu m}$ at 100 min (**e**) and 105 min (**f**) at stage III.

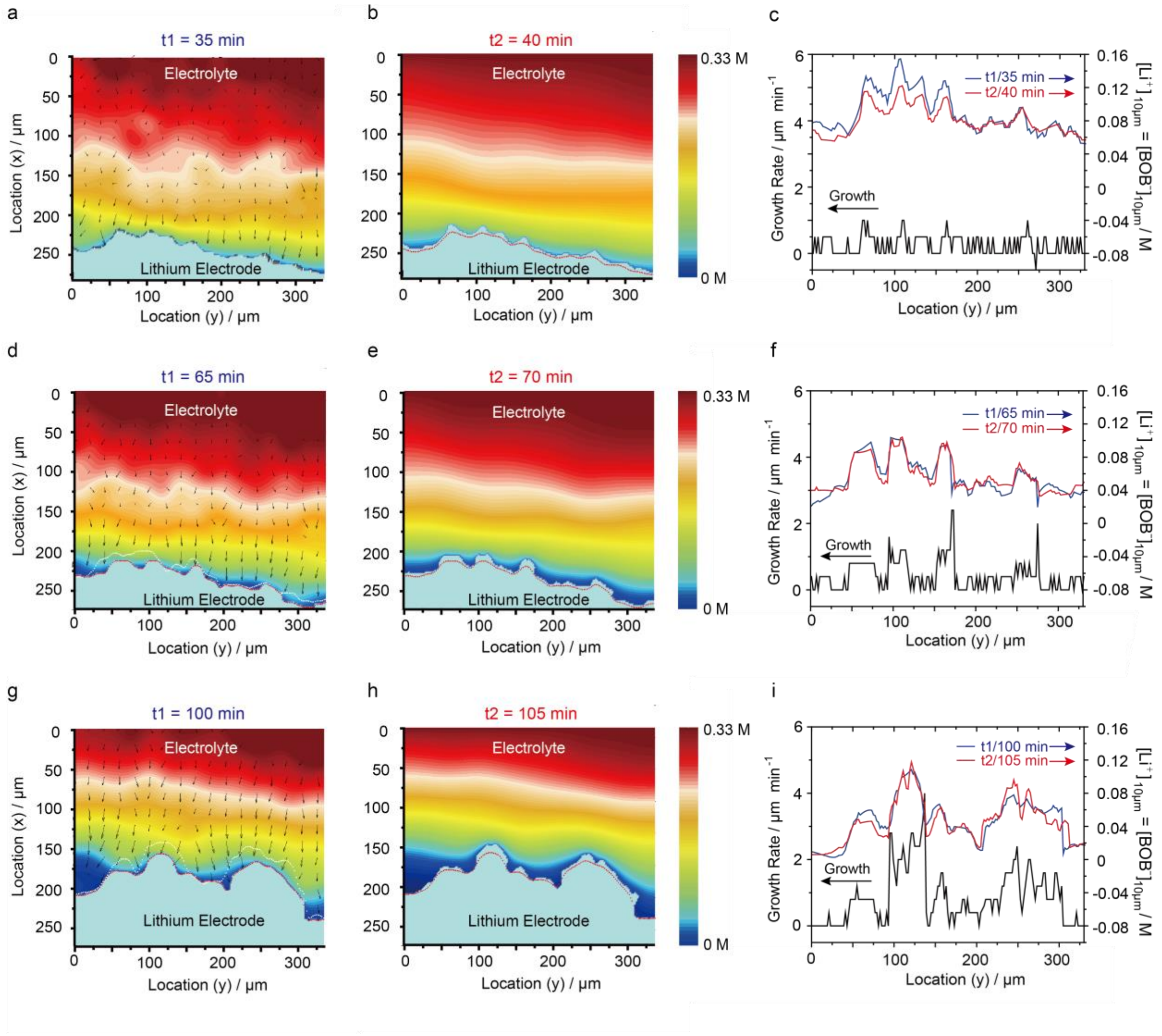

Supplementary Figure 14. Phase-field simulation results of Li growth, which generates results in Table 1. The first column exhibits the experimental concentration results and solid lithium electrode region at t1 (**a** / 35 min, **d** / 65 min & **g** / 100 min), which are obtained by SRS imaging and used for simulating data in the second and third columns. The second column exhibits simulation results of Li^+ concentration and solid lithium electrode at t2 (**b** / 40 min, **e** / 70 min & **h** / 105 min). Boundaries of the lithium electrode at t1 are also labeled as the red contour in **b**, **e**, and **h** for comparison. The color bar is at right to show $[Li^+]$. The third column (**c**, **f**, **i**) shows the relationship between simulated local lithium growth rate (black line) and $[Li^+]_{10\mu m}$ at t1 (blue line) / t2 (red line).

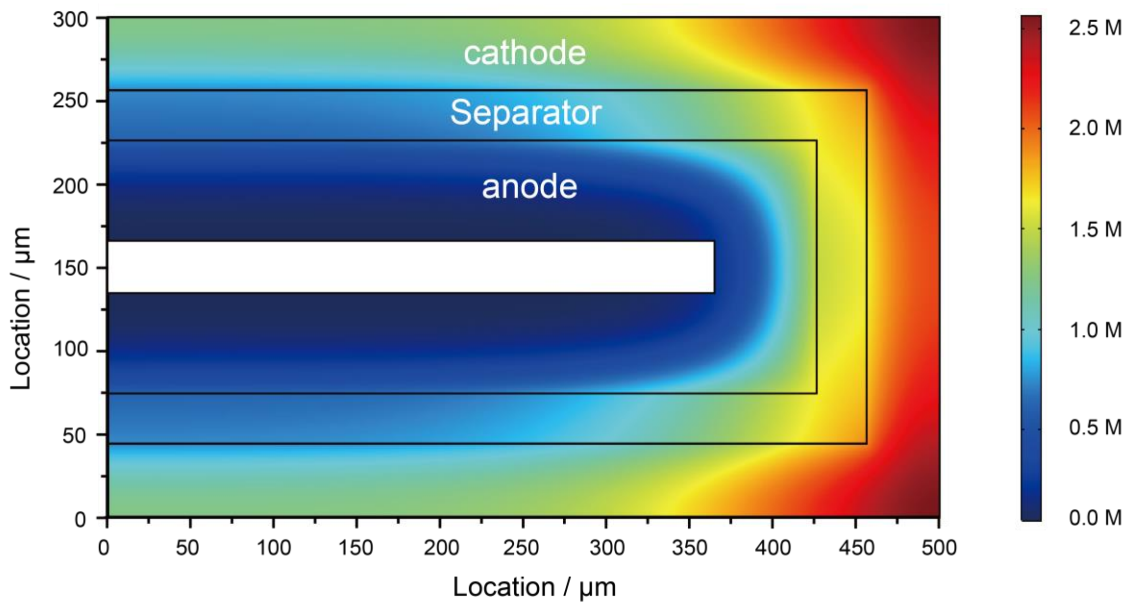

Supplementary Figure 15. A simulation of a commercial lithium-ion battery charged at 2C at room temperature, showing the depletion happening at the anode side. From inside to outside the component is porous anode / membrane / porous cathode. The cathode is $LiCoO_2$, the anode is graphite, and the electrolyte is 1M $LiPF_6$. The diffusion coefficient is set as 10^{-6} cm^2 s^{-1}. The cathode and the anode are also labeled as shown in the figure. We expect similar things to happen when porous lithium anode is used to replace graphite. Depletion may become worse at a lower temperature, which decreases the diffusion coefficient of Li^+ ions. The simulation is performed by COMSOL.

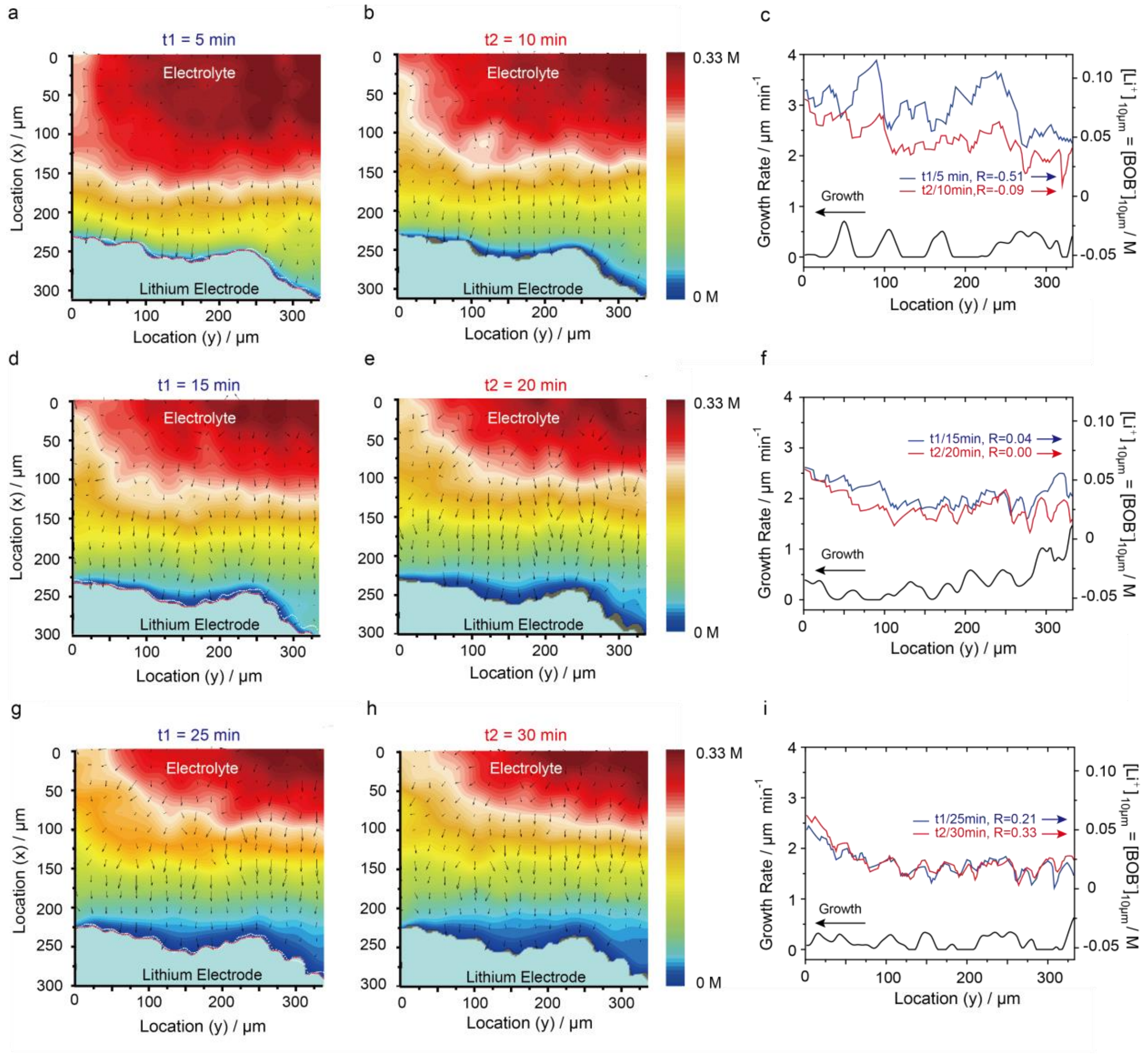

Supplementary Figure 16. Experimental 2D overlapped images at three representative moments of lithium electrode with Li_3PO_4 artificial SEI. **a–c** 5/10 min, **d–f**: 15/20 min, **g–i**: 25/30 min are picked to show Li^+ concentration profile and lithium growth. The first column exhibits the Li^+ concentration profile and solid Li electrode region at t1 (**a**/5 min, **d**/15 min & **g**/25 min). The boundaries of lithium electrode at t1 and t2 are labeled by red and white dash lines, respectively. The second column exhibits Li^+ concentration profile and solid Li electrode region at t2 (**b**/10 min, **e**/20 min & **h**/30 min). Color bars on the right show the Li^+ concentration. In **b**, **e** and **h**, the turquoise color is the area of lithium electrode at t1, and the brown part is the lithium grown between t1 and t2. Black Arrows are local Li^+ concentration gradient. The third column (**c**, **f**, **i**) shows the spatial distribution of local dendrite growth rate (black line) and local $[Li^+]_{10\mu m}$ at t1 (blue line) / t2 (red line).

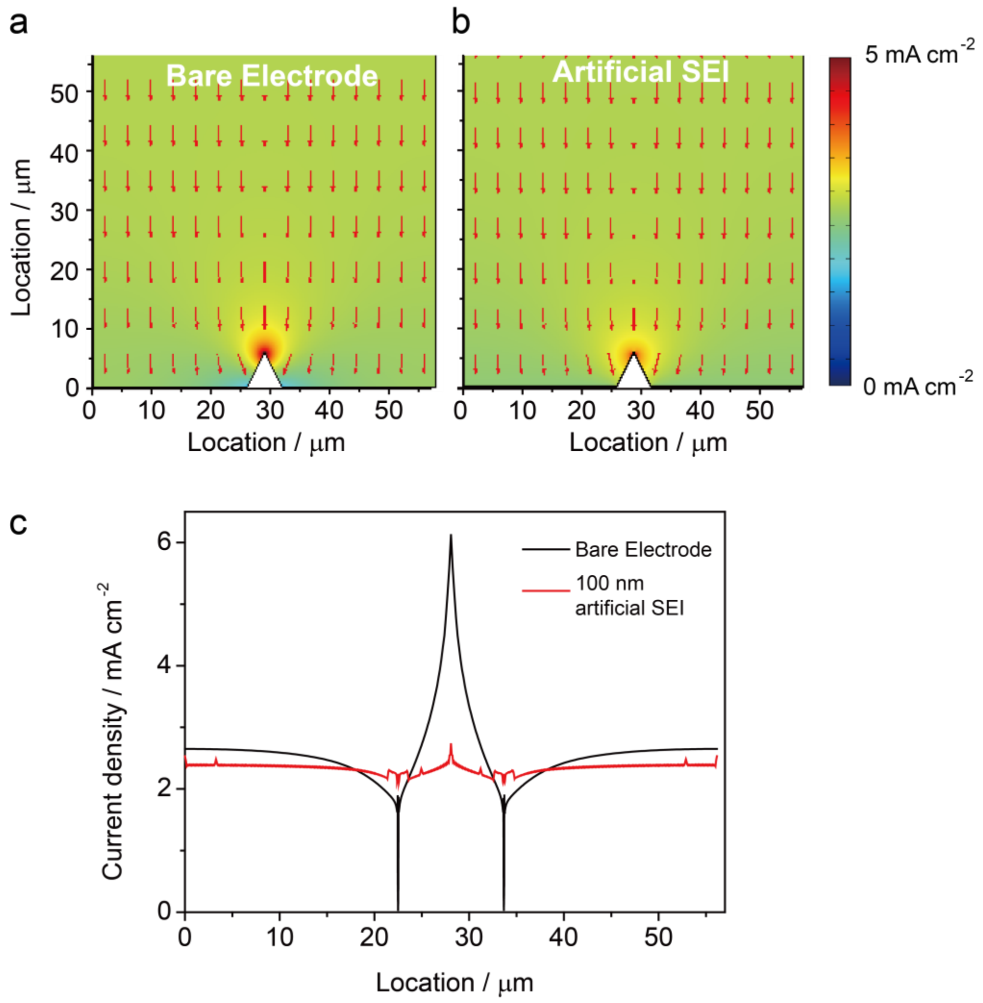

Supplementary Figure 17. A simulation of current density distribution near Li cathode in a Li/Li symmetric cell. The triangle tip mimics a single dendrite. **a** and **b**, current distribution on **a**. A bare lithium electrode, and **b**. A lithium electrode coated with a 100 nm artificial SEI. **c**. Current distribution at lithium surface of the two cells. The total currents for **a** and **b** are the same, both at 2.4 mA cm^{-2}. When SEI is applied, the heterogeneity of current density on the Li metal surface is greatly alleviated. The maximum current at lithium surface is reduced from 6.1 mA cm^{-2} for bare lithium (**a**) to 2.8 mA cm^{-2} with 100 nm SEI (**b**). In the simulation, the electrolyte has an ionic concentration of 1 M, and ionic diffusivity of 10^{-6} cm^2 s^{-1} for both cations and anions. In the coating layer, the transference number of Li^+ (t_+) is 0.99, and ionic diffusivity of Li^+ is 10^{-8} cm^2 s^{-1}.The relation between ionic diffusivity and mobility obeys the Einstein relation.

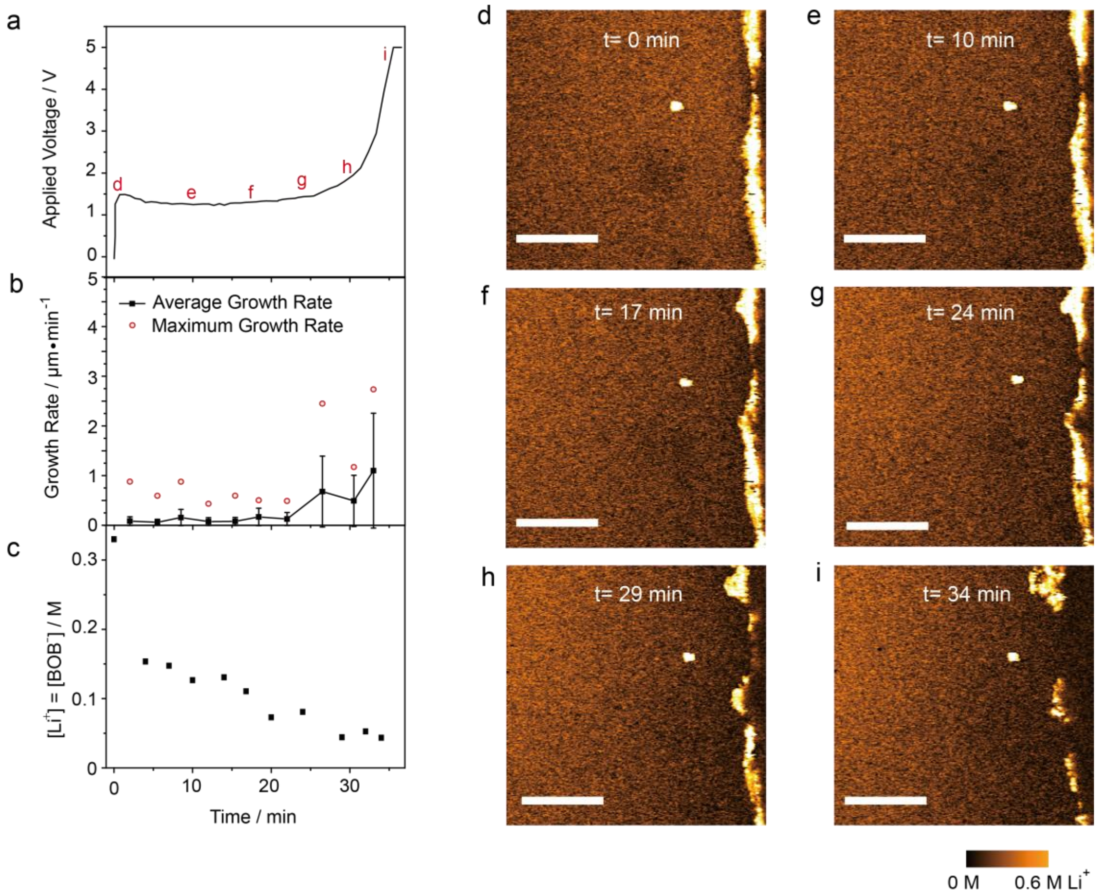

Supplementary Figure 18. The effect of adding an electrolyte additive TBA-PF_6. **a**. Voltage profile of a Li/Li cell with the addition of 0.05 M TBA-PF_6 showing the voltage change versus time, **b.** Lithium growth rate and standard deviation vs. time. Solid square is the average lithium growth rate and the red circle is the maximum growth rate. **c.** [BOB^-] change at 10 μm away from the electrode surface. **d–i** correspond to SRS 2D images at time 0 minute, 10 minutes, 16.8 minutes, 24 minutes, 29 minutes and 34 minutes. The current density is 1.45 mA cm^{-2}. Scale bars in **d–i** are all 100 μm.

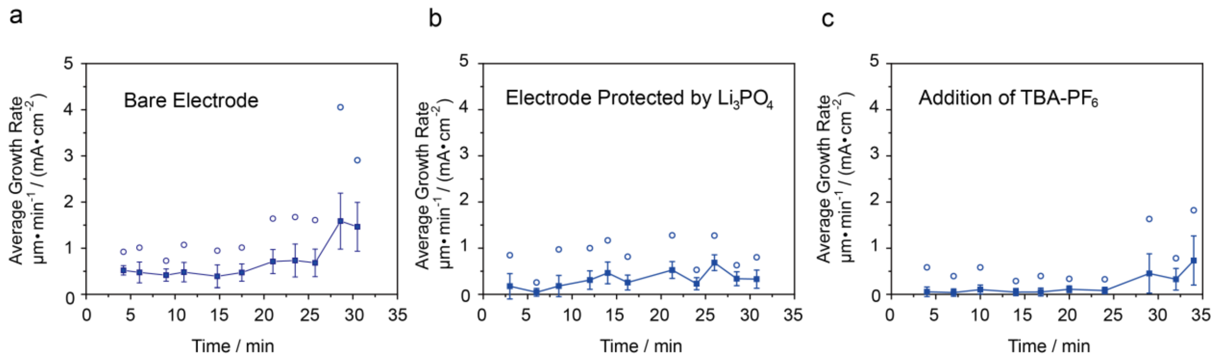

Supplementary Figure 19. Growth speed normalized by current density. **a**. Bare lithium (originally Fig. 3d). **b**. Li_3PO_4 artificial SEI-coated lithium (originally Fig. 5b). **c**. The addition of TBA-PF_6 (originally Supplementary Fig. 18b).

Supplementary Table 1. Diffusion data. Concentrations, conductivities, and the corresponding diffusion coefficients.

Concentration	Conductivity S cm^{-1}	Diffusion Coefficient cm^2 V^{-1} s^{-1}
0.1M	$3.6*10^{-4}$	$4.9*10^{-7}$
0.3M	$1.1*10^{-3}$	$4.7*10^{-7}$
0.5M	$1.8*10^{-3}$	$4.9*10^{-7}$
Fitted	/	$4.9*10^{-7}$

Supplementary Note 1. Theoretical Analysis on Electroneutrality in the electrolyte

In this paper, the electroneutrality does not mean the difference between [BOB^-] and [Li^+] is zero; the difference is small enough to be neglected at given resolutions of SRS microscopy. First, we consider a planar electrode:

According to Poisson's Equation for electrostatics, the electrical potential **Φ** and ionic distribution satisfy:

$$\nabla^2\Phi = -\frac{F}{\varepsilon\varepsilon_0}\sum_i z_i c_i \qquad (1)$$

Where F is the Faraday constant (96485 C mol^{-1}), ε is the relative permittivity of electrolyte and ε_0 is the vacuum permittivity (8.85×10^{-12} F m^{-1}), and z_i and c_i are charge number and concentration of all ions in the electrolyte. In our cell, the maximum voltage is 5 *V*, and the distance is around 500 μm. Even if we consider an extreme condition, in which the maximum gradient of an electrical field ($-\nabla^2\mathbf{\Phi}$) is 5V μm^{-2}, and relative permittivity is 10, the difference in anion and cation concentration ($C_+ - C_-$) is still < 5 μM. Therefore, Li^+ can be reflected by BOB^- outside the double layer region (Debye length is 48.7 nm for 5 μM LiBOB) which is much less than our spatial resolution.

Then a nano-electrode was also considered. We performed a simulation of ion distribution in such a Li/Li symmetric cell by Multi-Physics Comsol. The transference number is assumed to be 0.5 for both Li^+ and anion, the initial concentration is 1 M, the distance between two lithium electrodes is 100 μm, the diffusion coefficient of both Li^+ and anion is $5*10^{-7}$ cm^2 s^{-1}, and the current density is 2 mA cm^{-2}. The electrode is designed to have a single tip with a width of 10 nm and a length of 2 μm. Supplementary Fig. 2 shows the zoom-in image of the tip. In such a cell, when Li^+ ion is fully depleted at the tip (Supplementary Fig. 2a), the current density is mainly near the tips (Supplementary Fig. 2b) and the voltage decay more near the tip (Supplementary Fig. 2c). In this situation, the highest cation-anion concentration difference is observed at the electrode tip, which is still smaller than 0.1 mmol L^{-1} and can be negligible in our experiment (Supplementary Fig. 2d).

Prof. Henry White at University of Utah also analyzed the effectiveness of electroneutrality on microelectrodes[1]. Under voltammetric response and at a position outside the Debye length, the

local net charge in the electrolyte (Σ_{error}) near a spherical microelectrode satisfies

$$-r_0^2 \frac{\varepsilon\varepsilon_0}{F}\nabla^2\phi = 18.4nm^2mM[\frac{r}{r_0}+\frac{2\gamma(r/r_o)^2}{I/I_l}]^{-2} \equiv r_0^2(c_+ - c_-) \qquad (2)$$

Where r_o is the radius of spherical microelectrode, F is the Faraday constant (96485 C mol^{-1}), ε is the relative permittivity (ε=7.8 for TEGDME)[2] and ε_0 is the vacuum permittivity (8.85*10^{-12} F m^{-1}), **Φ** is the potential difference between the position r in the solution and the bulk of the solution, γ represents the ratio of supporting electrolyte to reactant concentration, I/I_l is current after being normalized by limiting current and, c_+ /c_- are concentrations of all cations and anions respectively.

Therefore, the maximum charge difference occurs at γ = 0, which means no supporting electrolyte to shield electrical field. This is also the same as our experimental condition, where the electrolyte is binary without supporting electrolyte (γ = 0). In this case, the second term in the square brackets is zero, and the equation can be simplified to

$$18.4(nm^2mM)r^{-2} \equiv (c_+ - c_-) \qquad (3)$$

Based on supplementary equation 3, the difference between [Li^+] and [BOB^-] is purely determined by the distance to the center of the microelectrode. Therefore, r could be much smaller in microelectrode than the bulk electrode. Even if the electrode size is 10 nm, and we measure 100 nm away from the electrode/electrolyte interface, $c_+ - c_-$ = 18.4 / (110^2) = 1.52 × 10^{-3} mM , which is much less than SRS resolution. Besides, the typical Debye length is ~ 5 nm at the concentration of 1 mM, so 100 nm satisfy the prerequisite of outside Debye region.

Supplementary Note 2. Experimental Analysis on Electroneutrality in the electrolyte

As the SRS setup at Columbia can only detect wavenumber higher than 1000 cm^{-1}, we cannot directly see Li^+ ion based on Li-electrolyte interaction. Therefore, we track both Li^+ and BOB^- near the electrode surface by spontaneous Raman to derive their local concentration. The electrolyte used in this experiment is LiBOB / (TEGDME: EC *v*/ 7:3) instead of LiBOB / TEGDME, so that $[Li^+]$ can be tracked by the Li^+-EC interaction at 725 cm^{-1}, and BOB at 1830 cm^{-1} (Fig. 2a).

As shown in the inset of Fig. 2a, the intensities at the designated wavenumbers have excellent linearity with concentration so that the derived $[Li^+]$ and $[BOB^-]$ have high accuracy. The standard deviation is determined to be 8.3 mM for Li^+-EC solvation and 5.8 mM for BOB^- for a 14-second accumulation per point. It should be noted that the accumulation time is 10^6 longer than SRS to get similar chemical resolution.

Then we built the same lithium-lithium electrolytic cell as Figure 1c in the main text to probe local ion concentration under spontaneous Raman (XploRA One by HORIBA). Then this electrolytic cell was tested under variant current densities (from the 0.5 mA cm^{-2} to 1.5 mA cm^{-2}, Supplementary Fig. 3a) until ions at electrode surface are fully depleted together with lithium dendrite growth. The changing current density can also help verify the electroneutrality under a different electrical field.

During the cell operation, lithium is deposited as dendrite and gradually approach the Raman spot which has a size ~10 μm. We find that when the laser directly shines at lithium, severe signal loss and distortion will occur, which may be due to interactions with SEI and lithium. Therefore, we only approach ~ 10 μm away from the lithium surface, which is similar to our paper (Fig. 2c).

After converting Raman intensity to ion concentration, we can see that $[Li^+]$ and $[BOB^-]$ are reduced due to ion depletion (Supplementary Fig. 3b & Fig. 2d). Their absolute concentrations synchronize with each other so that they appear to be the same as each other and electroneutrality is valid.

To further answer this question more quantitatively, we performed the hypothesis testing in statistics. Data at 50 minutes were used as an example here. For cations $X_C \sim N(\mu_C, \sigma_C^2)$, X_C is the measured cation concentration 0.01532 M and σ_C is the standard deviation of cation concentration 0.00828 M. The same goes with anions, $X_A \sim N(\mu_A, \sigma_A^2)$ while X_A = 0.01455 M

and σ_A = 0.0058 M. We want to test whether the mean concentration for cations μ_C and anions μ_A are consistent. The null hypothesis is $\mu_C = \mu_A$ with a significance level of 1%.

$X_C - X_A$ is normally distributed with a mean of $\mu_C - \mu_A$ and standard deviation $\sqrt{\sigma_C^2 + \sigma_A^2}$. For hypothesis $\mu_C - \mu_A = 0$:

$$\frac{X_C - X_A - 0}{\sqrt{\sigma_C^2 + \sigma_A^2}} \sim N(0,1) \tag{4}$$

The test statistic is 0.074, far smaller than $Z_{0.005}$ ($Z_{0.005}$ =2.58, the value can be found in Z table), so the null hypothesis is accepted. Other experimental results (50 min – 65 min) with test statistics (–0.363, 0.082, –0.216, 0.148) all fall in the range from $-Z_{0.005}$ to $Z_{0.005}$.

It is worth noticing that the sum of the square of each time also follows a chi-square distribution. The chi-square test statistics is $\sum\left(\frac{X_C - X_A}{\sqrt{\sigma_C^2 + \sigma_A^2}}\right)^2$ =0.182, which is much smaller than the chi-square table at the degree of freedom of 5 at the significance level of 5% (16.75). We can conclude that the concentration of cation and anion are equal in the whole experiment.

Supplementary Note 3. Effect of salt-solvent Interaction

There are usually numerous ion complexes that are also present in the electrolyte, such as solvent-separated ion pair (SSIP), contact ion pair (CIP) or aggregate (AGG) solvates in which the anions are coordinated to zero, one and two or more Li^+ cations, respectively[3]. However, based on Supplementary note 1 & 2, Electroneutrality is valid in all battery electrolytes, and the difference between [Li^+] and [anion] is much less than the resolution of SRS (1–10 mM), no matter whether the interaction exists.

The critical question is that if Raman signal is proportional to [Li^+] and [anion] with the existence of ion complexes as Raman peaks may split or distort due to ion interaction. First, we have experimentally observed high linearity in anion concentration vs. Raman intensity in LiBOB/TEGDME, LiTFSI/TEGDME, and LiTFSI/ DMC up to high concentrations (Supplementary Fig. 4). In LiBOB/TEGDME, it is 0–0.5 M as the solubility is ~ 0.7 M, and it is 0–2 M for LiTFSI in TEGDME or DMC. The Raman intensity is for a given wavenumber instead of peak integration, to make it consistent with SRS measurement. Both correlation coefficients are higher than 0.999. We indeed need to identify these modes, to see whether CIP/AGG is involved, but no matter whether it is for free or with interaction, experimental Raman signal is linear with anion concentration in a reasonable range for battery electrolyte, as shown in Supplementary Fig. 4. Second, strong interaction to cause nonlinearity only occurs for really high concentration, which is not common in battery systems[3], and we are studying low ion concentration in depletion, so linearity should be general in this range. Third, the Raman intensity of direct Li^+-solvent interaction is linear with Li^+ concentration as shown in Fig. 2a, the intensities of Li^+-EC have excellent linearity with Li^+ concentration.

Therefore, the salt-solvent will not generate deviation in SRS experiments.

Supplementary Note 4. Wavenumber range of SRS microscopy

In the current configuration, the Stokes laser is fixed at 1064 nm, and a pump laser could be tuned in principle from 720 to 990 nm (corresponding to 700 – 4500 cm^{-1}). Due to the currently optimized stability for OPO tuning, the optimal detection window ranges from 1000 to 3300 cm^{-1}. Therefore, BOB^- (1830 cm^{-1}) is chosen over PF_6^- (770 cm^{-1})[4] and Li-propylene carbonate (721 cm^{-1})[5]. The detection range can be expanded by further optimizing the OPO cavity to stabilize the tuning range around 700 cm^{-1} for monitoring the counter ions as mentioned above.

Supplementary Note 5. Detection limit of ionic concentration

The detection limit is determined from the concentration curve shown as the inset of Fig. 1d when the SRS signal-to-noise ratio equals to 1. The signal is linearly extrapolated from concentration curve, while the noise is read from the standard deviation of pump-only SRS image. Such noise level in our experiment is approaching the laser shot-noise level. The ionic concentration limit is finally determined to be 10 mM. Dwell time: 2 μs per pixel, 16-time frame average. Power: P_{pump} = 24 mW and P_{pump} = 50 mW on sample.

Supplementary Note 6. Simulation

The simulation follows previous Newman's model[6]. The transport of lithium ion can be solved by 1D Nernst-Planck equation:

$$N_i = -z_i F \mu_i c_i \nabla \phi - D_i \nabla c_i \tag{5}$$

In this equation, N_i is the flux density of species i; $z_i F$ is the charge per mole on the species. D_i and μ_i are the diffusion coefficient and migration coefficient of species i, respectively. The first term on the right side represents the ionic flux under the electrical field; the second term represents the diffusion current. Convection is neglected since the gel immobilizes the electrolyte. By combining Nernst equation of both Li^+ and BOB^-, and cancelling the migration term, the ion transport behavior can be reduced to

$$\frac{\partial c_{Li^+}}{\partial t} = D^* \frac{\partial^2 c_{Li^+}}{\partial x^2} \tag{6}$$

$$D^* = \frac{\mu_{Li^+} D_{BOB^-} + \mu_{BOB^-} D_{Li^+}}{\mu_{Li^+} + \mu_{BOB^-}} \tag{7}$$

and the boundary conditions are:

$$\frac{j}{F} = \frac{D^*}{1-t_+} \frac{\partial c}{\partial x} \quad \text{at } x=0 \text{ and } x=L \tag{8}$$

j is the current density applied to the cell, and L is the distance between two lithium electrodes. This equation is used to fit experimental results in Fig. 3 and the results are labeled as red lines in Fig. 3g. D is simulated based on least squares estimator. Here t_+ is assumed as 0.5 for convenienc[7, 8]. The details can be found in chapter 11 of Electrochemical Systems authored by Prof. John Newman[6].

Supplementary Note 7. Experimental measurement of ionic diffusion coefficient

The effective ionic mobility based on experimental ionic conductivity and ion concentration according to the equation

$$\sigma = F\sum c_i \left|z_i\right| \mu_i \quad (9)$$

Where σ is the conductivity of gel electrolyte obtained from Electrochemical Impedance Spectroscopy (EIS) measurements. μ is the ionic mobility. z_i is +1 for Li^+, and –1 for BOB^- with the assumption that $\mu_+ = \mu_-$, μ of Li^+ can be derived. Then the diffusion coefficient is calculated based on Einstein relation:

$$D = \frac{\mu k_B T}{e} \quad (10)$$

The data can be found in supplementary table 1.

Supplementary Note 8. Current stepping test

Current stepping test further confirms the ion depletion-driven dendritic growth in a different Li/Li cell (Supplementary Video 2 & Supplementary Fig.11). When 0.6 mA cm^{-2} is applied, which is smaller than limiting current (0.75 mA cm^{-2}), the Li^+ concentration at Li surface is stabilized at 0.1 M after 62 minutes, and the growth rate of Li is also steady (v_{ave} ~ 0.2 μm min^{-1}). Once the current is increased to 0.9 mA cm^{-2}, voltage increases to 2.8 V due to electrolyte resistance and charge transfer overpotential, but still keeps climbing up as a result of depleted ion concentrations. The surface concentration of Li^+ decreases quickly to 0 in 20 minutes. Meanwhile, the lithium growth rate shows a clear jump to v_{ave} =1.14 μm min^{-1} (65/70 Stage *II*) and finally to v_{ave} =1.62 μm min^{-1} (100/105 Stage *III*). Such observation demonstrates that the growth rate of Li dendrite depends on local Li^+ concentration.

Supplementary Note 9. Moving speed of ions in the gel electrolyte

The speed of ion (*v*) can be deducted from

$$j = 2Fcv \tag{11}$$

Where *c* is the ion concentration, and 2 comes from the assumption that Li^+ and BOB^- move at the same speed. When Li^+ concentration is 10 mmol L^{-1} and the current density is 0.9 mA cm^{-2} (as in Figure 4), *v* is 4.5 μm s^{-1}.

Supplementary Note 10. Phase-Field Simulation

The Li dendrite morphology evolution and Li-ion diffusion during the selected electrodeposition periods (35–40, 65–70, 100–105 min) were simulated by a phase-field method in two-dimension. A phase-field order parameter, $\phi(r)$, continuously varying from 1 to 0, was defined spatially to distinguish the Li metal ($\phi = 1$) from dilute liquid electrolyte ($\phi = 0$) with a finite thickness of diffuse interface at the phase boundary. The diffusion-limited reaction $Li^{+} + e^{-} \rightarrow Li(s)$ takes place at the electrode / electrolyte interface with the assumptions that excess electrons are always supplied at the electrode surface (the activity of electron equals 1). The phase-field temporal evolution incorporating non-linear Butler-Volmer kinetics follows,

$$\frac{\partial \phi}{\partial t} = -L_{\sigma}(g'(\phi) - \kappa \nabla^2 \phi) - L_{\eta} h'(\phi) \left\{ exp\left[\frac{(1-\alpha)F\eta_a}{RT}\right] - c_{Li^+} exp\left[\frac{-\alpha F \eta_a}{RT}\right] \right\} \quad (12)$$

where $g(\phi) = W\phi^2(1-\phi)^2$ is the double well function, separating the two equilibrium states (Li metal at $\phi = 1$, and electrolyte at $\phi = 0$.) with an energy barrier height of $W/16$. $\kappa(\theta) = \kappa_0[1 + \delta\cos(\omega\theta)]$ is the gradient coefficient with the interface energy-related coefficient κ_0, anisotropy strength δ, and crystalline symmetry mode ω ($\omega = 4$ for body-center cubic Lithium metal). The interpolating function $h(\phi) = \phi^3(6\phi^2 - 15\phi + 10)$ confines the exponential electrochemical driving force within the electrode / electrolyte interface region. η_a is the activation overpotential. c_{Li^+} is the local concentration of Lithium ion. α is the charge-transfer coefficient ($\alpha = 0.5$ in this study). R, T, and F are gas constant, the temperature of the system and Faraday's constant, respectively. The Li-ion diffusion is described by Nernst-Plank equation, and the electrostatic potential distribution is governed by Poisson equation, which was coupled and solved together with the phase-field evolution equation (6). The phase field model and Li electrodeposition parametric details are in reference[9, 10].

All phase-field simulations were performed by COMSOL Multiphysics with finite element meshing. To be consistent with the SRS results, a 320 × 320 μm simulation size with the same electrolyte bulk concentration (0.33 M) and *I-V* boundary conditions were adopted. The experimental Li dendrite morphologies and Li-ion concentration distribution profiles at 35, 65 and 100 min were used as initial phase-field inputs and simulated for 300 s, respectively.

Supplementary References

1. Smith, C. P. Theory of the voltammetric response of electrodes of submicron dimensions. Violation of electroneutrality in the presence of excess supporting electrolyte. *Anal. Chem.* **65**, 3343–3353 (1993).

2. Wohlfarth, C. *Static dielectric constant of pure liquids and binary liquid mixtures* (Springer, 2008).

3. Seo, D. M., Borodin, O., Han, S.-D., Boyle, P. D., Henderson, W. A. Electrolyte solvation and ionic association II. Acetonitrile-lithium salt mixtures: highly dissociated salts. *J. Electrochem. Soc.* **159**, A1489–A1500 (2012).

4. Chagnes, A., Carré, B., Willmann, P., Lemordant, D.. Modeling viscosity and conductivity of lithium salts in γ-butyrolactone. *J. Power Sources* **109**, 203–213 (2002).

5. Kondo, K.*, et al.* Conductivity and solvation of Li^+ ions of $LiPF_6$ in propylene carbonate solutions. *J. Phys. Chem. B* **104**, 5040–5044 (2000).

6. Newman, J., Thomas-Alyea, K. E. *Electrochemical systems* 3rd edn (John Wiley & Sons, 2012).

7. Bai P., Li, J., Brushett, F. R., Bazant, M. Z. Transition of lithium growth mechanisms in liquid electrolytes. *Energy Environ. Sci.* **9**, 3221–3229 (2016).

8. Bruce, P. G., Vincent, C. A. Steady state current flow in solid binary electrolyte cells. *J. electroanal. chem. interfacial electrochem.* **225**, 1–17 (1987).

9. Chen, L.*, et al.* Modulation of dendritic patterns during electrodeposition: A nonlinear phase-field model. *J. Power Sources* **300**, 376–385 (2015).

10. Liu, Z., Qi, Y., Lin, Y., Chen, L., Lu, P., Chen, L.. Interfacial study on solid electrolyte interphase at Li metal anode: implication for Li dendrite growth. *J. Electrochem. Soc.* **163**, A592–A598 (2016).
