# Supplementary material for: Operando and three-dimensional visualization of anion depletion and lithium growth by stimulated Raman scattering microscopy
Source: Nat Commun. 2018 Jul 30;9:2942. doi: 10.1038/s41467-018-05289-z (PMC6065384; doi:10.1038/s41467-018-05289-z)
Supplement: Supplementary file 3 — Description of Additional Supplementary Files [file 41467_2018_5289_MOESM3_ESM.pdf]

Description of Additional Supplementary Files

File Name: Supplementary Video 1

Description: Visualization of ion transport / depletion and dendrite growth on a bare 2D Li electrode. This video corresponds to Fig. 3. In the video, the upper part is the voltage profile, the lower left and lower right are the top view and the side view of reconstructed 3D SRS images respectively. The voltage profile and SRS 3D images are synchronized for intuitive understanding. The length is 424 μm.

File Name: Supplementary Video 2

Description: Video that shows the correlation between local Li growth and local Li^+ concentration. This video corresponds to Fig. 4. In the video, the upper part is the voltage profile; the current changes from 0.6 mA cm^{-2} (lower than limiting current) to 0.9 mA cm^{-2} (higher than limiting current) at 62 minutes. The lower left and lower right are the top view and the side view of reconstructed 3D SRS images respectively. The voltage profile and SRS 3D images are also synchronized. The length is 509 μm.

File Name: Supplementary Video 3

Description: Visualization of ion concentration profile and dendrite growth on a Li electrode protected by Li_3PO_4. This video corresponds to Fig. 5. The video clearly shows that under the protection of artificial SEI, the dendrite will not thrive regardless of the Li^+ depletion. The upper part is the voltage profile; the lower left and lower right are the top view and the side view of reconstructed 3D SRS images respectively. The voltage profile and SRS 3D images are also synchronized. The length is 509 μm.
